# Practical Medicine

**Published:** 1879-03

**Authors:** 


### Summary.

*Collaborators:*

DR. H. GRADLE, DR. L. W. CASE, DR. R. PARK,
DR. R. TILLEY, DR. D. R. BROWER.

### PRACTICAL MEDICINE.

TURPENTINE IN WHOOPING COUGH. (*Wiener Allegem. Med. Zeit.*, No. 12, 1878.)

Dr. Gerth cured a case of laryngeal catarrh by placing twenty drops of turpentine on a handkerchief, held before the face and causing about forty deep inspirations to be taken. Repeating this thrice daily, the cure was quite rapid. In the same family he found an infant fifteen months old in the convulsive stage of whooping cough, quite exhausted, and vomiting all ingesta. There was at the same time slight bronchial catarrh with slight evening rise of temperature. Gerth decided to experiment here also with turpentine. He directed the mother to hold the moistened cloth as above, before it when awake, and to drop the oil upon its pillow when asleep. The result was most happy. Within the twenty-four hours the frequency and severity of the attacks notably diminished. The child's strength was sustained by stimulants, and improvement was very rapid. Within a year pertussis became epidemic in his vicinity, and he repeatedly tested the drug in this way. He gave it to children of all ages, and in any stage of fever. The initial catarrh, the convulsive, and the final catarrhal stages were all decidedly benefited, the spasmodic attacks being in many cases aborted.

